# Learning through clinical extramural studies: an observational study

**DOI:** 10.1186/s13620-023-00238-9

**Published:** 2023-06-09

**Authors:** Diane Cashman, Sue Rackard

**Affiliations:** 1 Veterinary Education, School of Veterinary Medicine, University College Dublin, Dublin 4, Ireland; 2 Associate Dean for Teaching and Learning in the School of Veterinary Medicine, University College Dublin, Dublin 4, Ireland

**Keywords:** Clinical extramural studies, Workplace learning, Self-regulated learning

## Abstract

**Background:**

Veterinary medicine programmes require students to learn in formal educational settings and through workplace experiences. Previous studies have indicated that learning in the clinical workplace can be informal as students participate in daily activities of service provision by veterinary teams. It can be complex however for students to transition from a traditional formal educational setting to learning in the workplace and students must be able to self-regulate their learning. This requires students to set their own learning goals, consider available learning opportunities and to evaluate if intended learning outcomes have been attained. There is a need to identify strategies students undertake to self-regulate their learning in the workplace to design supports to enhance their learning. The aim of this study was to provide a detailed description of how final year veterinary medicine students plan, learn and reflect on their learning in the workplace context of clinical extramural studies (CEMS) prior to the COVID-19 pandemic.

**Methods:**

An observational repeated cross-sectional design study was conducted with two groups of final year veterinary medicine students in University College Dublin. Data was collected in two stages by analysing student activity records and surveying students in 2017 and 2018. Participants were asked to describe how they planned their CEMS, to describe the types of learning activities they participated in, and describe their reflections of CEMS.

**Results:**

The results are interpreted through the lens of self-regulated learning theory. Analyses of student CEMS activity records indicate that students from both groups primarily participated in small animal / production animal or mixed practice work placements. The majority of respondents of the survey indicated that CEMS was a valuable learning opportunity and they were motivated by placements that would support their future career goals. Financing CEMS placements was a key obstacle to their planning. The majority of respondents indicated varying frequencies of engaging in different types of learning activities and noted that finding suitable placements that facilitated practical skill development and active student learning was a challenge. Implications for veterinary education are discussed.

**Conclusions:**

Student perspectives on planning and learning in the CEMS workplace context yielded important insights into the factors that influence their self-regulatory activities which can help inform future educational interventions to support student learning.

## Background

Workplace learning (WPL) is an important and essential component of a veterinary medical programme as it offers students the opportunity to learn in an authentic real-life work environment, which can assist them to develop their identity as they transition into the veterinary profession [1, 2]. It can be delivered through diverse settings that include off-campus experiences undertaken on farms, first opinion veterinary practices, or through on-campus intramural experiences in a referral hospital setting. Scholz [3] argues that WPL if considered through a social practice lens can provide students with opportunities to learn about the complex interrelationships that occur in veterinary practice. WPL can support students' appreciation of veterinary professional working life, guide their future career choices [4], help them to develop their competencies to manage challenging situations [5], and to apply disciplinary knowledge acquired during formal education [6].

Learning in the workplace however can be challenging for students as veterinary practices need to prioritise services and everyday work activities, therefore learning can become complex and multimodal [7]. Much of the current literature on workplace learning pays particular attention to its informal and opportunistic nature [8-11]. Eraut [8] explains there are four main types of workplace activities that support learning: participation in group activities, working with colleagues, tackling challenging tasks, and working with clients; and highlights that outcomes can depend on contextual factors (allocation of work, relationships with people at work, expectations of role) and learning factors (challenge, feedback and support, confidence and commitment). These activities can be challenging to facilitate given the role of the clinical teacher in the workplace who must balance student learning and patient welfare [12].

To overcome the challenges of WPL, an important educational component for a veterinary medicine curriculum is to foster students' ability to self-regulate their learning (SRL) to be able to capitalise on the informal learning opportunities available in a work context [13, 14]. According to Zimmerman [15] SRL is defined as “*the self-generated thoughts, feelings, and actions that are planned and cyclically adapted to the attainment of personal goals*”, (p14). The literature in SRL highlights several phases and constructs that encompass a learner's ability to regulate their learning: planning, learning, assessment and adjustment [16], and several models of SRL have been developed to explain this complex learning process [17]. White’s model of SRL [16] describes how students set their own learning goals during the planning SRL phase. These goals can be influenced by multiple factors such as the student’s confidence and belief in their abilities, and the social environment through which learning takes place. During the learning phase of SRL students seek out learning opportunities to reach their intended goals and they will adjust their learning strategies if required. Students monitor and assess their learning progress during the assessment phase, determining if goals are reached by seeking external feedback where necessary or through self-monitoring processes. These activities will inform the student’s adjustment SRL phase by helping them to critically reflect on past learning experiences and to modify future learning plans. These processes encompass the metacognitive, cognitive and behavioural aspects of learning [18]. The development of SRL is considered an element core to development of lifelong learning skills [19] a day-one competency defined by regulatory bodies of veterinary medical programmes worldwide [20, 21] and framework for veterinary competency-based education [22]. The veterinary profession is continuously changing and practitioners are required to keep up to date in medical advances, diagnostic techniques and patient care to meet stakeholder and society's needs [23]. Veterinary students must develop their SRL processes to ensure they are able to continue and succeed in their journey of lifelong learning [24]. CEMS provides a valuable opportunity for students to commence this process at an early stage of their veterinary education.

In this context, there is a need to examine what activities students undertake to plan their CEMS and learn while on placements in order to develop evidence-based interventions to support their learning and development of SRL competence.

### Study context

At University College Dublin (UCD), School of Veterinary Medicine students on the five-year school-leaver (MVB) and four-year Graduate Entry (GE) veterinary medicine programmes undertake 24 weeks of work placements outside of UCD, referred to as CEMS, in the clinical years of their programme. CEMS is a co-curricular activity and a programme requirement that aims to support their studies, where in the final year they complete intramural rotations in the University Veterinary Hospital or in first opinion practices (e.g. The Dublin Society for Prevention of Cruelty to Animals). WPL has a long tradition in veterinary education in Ireland, United Kingdom and Australasia where it is known as CEMS and in Europe where it is most commonly known as *external practical training*. In Ireland, students must complete 11 core weeks in different practice types, for example small animal practice and equine practice. The remaining 13 weeks can be completed in any combination of the student’s choosing. Students are encouraged to consider a variety of veterinary sectors of employment to experience different learning opportunities, for example undertaking placements in a laboratory, contributing to a research project or attending conferences. Placements must be completed outside of the UCD academic trimester during Christmas, Spring and Summer breaks.

The aim of this study was to describe where final year students at UCD planned their CEMS placements, and to investigate their planning and learning strategies, and reflections about CEMS. The objectives of the study were to:

1. Examine the types of placements students completed for CEMS;
2. Describe strategies students adopted to plan their CEMS and what obstacles they faced;
3. Describe the frequency of engaging in learning activities on CEMS;
4. Describe student self-assessment of how CEMS supported the development of their day-one competencies and their future employability and their reflections on CEMS overall.

This study was conducted as part of an educational design research project [25] being completed for a doctoral thesis that is investigating interventions to promote students SRL on CEMS. Data was collated prior to the emergency transition of higher education to online learning due to the COVID-19 pandemic in 2020. CEMS was subsequently impacted by lock-down measures directed by government public health guidelines, for example the number of CEMS weeks was reduced and various concessions were made for student learning. This study gives insights into student learning on CEMS before the COVID-19 pandemic occurred, therefore it does not explore the impact of COVID-19 on student learning on CEMS.

## Method

An observational repeated cross-sectional study design was conducted with final year veterinary medicine students in UCD from the MVB and GE programmes in 2017 and 2018. Characterised by Cohen [26] a cross-sectional study provides a snapshot of a population at either one or two points in time. A repeated cross-sectional design [27] facilitated the retrospective exploration of student CEMS experiences with two independent samples over a two-year period. Data for this study was gathered using two methods, first student CEMS activity records were analysed to describe how students planned their CEMS placements by type, second an online student survey was conducted to explore SRL activities on CEMS. This study met the criteria exempting it from full ethical review from the UCD Human Research Ethics Committee—Sciences (Exemption Reference Number LS-E-17-Cashman-Doherty).

Student CEMS records from 2017 and 2018 were analysed in Microsoft Excel [28] (v15.4). Records were de-identified by replacing direct identifiers with a unique random identifier. A questionnaire was developed in an online format using SurveyMonkey [29] for ease of distribution and data collection. All final year students registered to the MVB and GE programmes were invited to participate in the study by email in March 2017 and March 2018 with no incentivisation. Two reminder emails were sent. Information regarding the aims of the study and participant confidentiality was provided. All responses were voluntary and anonymous. Analysis of the survey data was conducted on the aggregated data set from both samples. SPSS Statistics (v26) [30] was used to screen the data and to conduct descriptive statistical analyses of the quantitative data. Qualitative responses were hand coded using Microsoft Excel and thematic analysis was used [31, 32] to categorise the responses into themes.

### Questionnaire instrument

The questionnaire was adapted with permission from a previous survey conducted by the Royal College of Veterinary Surgeons (RCVS) with students in the UK about their EMS experiences in 2014 [33]. This questionnaire contained questions regarding planning and self-assessment processes of SRL that were adapted and expanded upon to meet the aims of our survey. A key difference between both questionnaires was that RCVS collated data for pre-clinical and CEMS experiences while our survey would only focus on CEMS. The RCVS questionnaire asked questions relating to how students booked their EMS, reasons why all placements of choice were not found and the location of placements. These questions were adapted into our questionnaire to explore the planning phase of CEMS. The RCVS questionnaire aimed to explore how EMS learning experiences related to professional skills, knowledge and clinical skills compared to their core university studies. While our survey did not seek this comparison the Likert-scale statements were used and developed in our questionnaire to capture student SRL learning activities on CEMS and their overall reflections on how CEMS supported the development of their day-one competencies. Specifically, our questionnaire contained five sections that contained Likert-scale and open-ended questions were designed to collect data regarding demographics and four phases of SRL: planning, learning, assessment and adjustment.

1. Demographics: Programme of study, age, gender, and home country.
2. Planning CEMS: Rate on a five-point Likert scale the factors that influenced their choice of placements; indicate how they identified placements from a ten-item list (strategies not identified could be recorded in an open-ended question), indicate from a list what obstacles (if any) prevented them from doing any placements; indicate how they financed different placement types.
3. Learning Activities on CEMS: Rate on a five-point Likert scale how frequently they participated in a list of learning activities on placement. These activities incorporated processes related to multiple SRL phases, for example discussing learning goals, observing a veterinary practitioner, seeking guidance, reflecting on previous tasks.
4. Assessment of Learning Experiences: Rate on a five-point Likert scale how they perceived CEMS supported the development of day-one competencies and understanding of veterinary professional life; 17 statements were provided for participants to self-assess their learning on CEMS.
5. Reflections of CEMS: Rate on a five-point Likert scale six statements regarding the perceived value of CEMS to support employability and relevance to their studies, indicate their overall experience of planning their placements. An open-ended question sought feedback on how CEMS could be improved.

Additional questions were asked of participants who indicated they completed a conference, laboratory or research placement; a further four questions sought data regarding the procedures, species and techniques seen on CEMS only, and opinions regarding the ePortfolio technology used for CEMS. Due to the size of the dataset those results are not reported in this paper but will be reported in a doctoral thesis.

## Results

### CEMS student activity records

Student CEMS records were analysed from two final-year student cohorts in 2017 (MVB *n* = 84, GE *n* = 38) and 2018 (MVB *n* = 71 and GE *n* = 23). Some records were excluded from analysis (2017 *n* = 1; 2018 *n* = 4) due to individual student registration circumstances, for example a student taking a year out. Table 1, shows how students allocated their weeks across these requirements noting 54 students in 2017 and 55 in 2018 completed more weeks than the required minimum of 24. The average number of weeks completed by students in 2017 was 25, the maximum undertaken was 32 weeks, compared to 2018 the average was 24.8 weeks and maximum 30 weeks. The majority of weeks completed by MVB students in 2017 (39.3%) and 2018 (38.6%) was in production animal / mixed practices. In 2017 over 40% of GE students completed their placements in small animal practices compared to 45.2% in 2018. The GE cohort completed more placements (2017 = 2.1%; 2018 = 3.7%) that were categorised with a primary focus on exotics compared to the MVB students (2017 = 0.9%; 2018 = 0.3%).

**Table 1:** Percentage of weeks spent by students on CEMS by placement type

Year	Programme	Number of student records analysed	Small Animal Practice	Production Animal /Mixed Practice	Equine Practice	Meat Plant	Laboratory	Exotics	Other	Total number of CEMS weeks completed by students
2017	MVB	84	33.8%	39.3%	16.6%	4%	1.9%	0.9%	3.5%	2097.7
2017	GE	38	41.8%	25.7%	14.7%	4.1%	4%	2.1%	7.8%	936.4
2018	MVB	71	33.7%	38.6%	17.3%	4%	2.8%	0.3%	3.2%	1781.8
2018	GE	23	45.2%	25.6%	11.8%	4%	1.6%	3.7%	8.2%	575

### Survey

There were 35 responses to the questionnaire from 128 students who were registered to final-year in 2017, while 48 responses were received in 2018 from 104 registered final-year students. Questionnaires that were less than 50% complete were excluded from analysis. Therefore 30 responses from the 2017 group and 42 responses from the 2018 group were analysed representing a response rate of 23% in 2017 and 42% in 2018. Data from both samples were merged and analysis was conducted on the aggregated data set. The final study sample consisted of 72 participants (69% female, 1 respondent did not disclose) with 72% aged between 20–24 years. 78% were registered to the MVB 5-year programme and 74% indicated their country was Ireland, while 15% noted Canada or USA.

## Planning CEMS

### Factors influencing CEMS placement choices

The majority of respondents rated four factors as *important* or *very important* when planning their CEMS, these included placements that: (i) may be beneficial to their future veterinary career (94%), (ii) provided experience of a discipline of possible future employment (94%), (iii) were affordable to complete (83%), and (iv) build a professional network (62%). 50% of respondents rated it was either *important* or *very important* that placements were located close to home. The results showed that selecting placements that support specialisation post-graduation was varied, 40% rated this factor as *important* or *very important,* while 22% indicated this was not *important.* Responses varied regarding travelling abroad as a factor that influenced their placement choice, 29% were neutral about this factor, while 39% considered this either *important* or *very important*, 32% considered this *not important* or *somewhat important.* Similarly, responses to complete placements as quickly as possible varied, 55% rated this factor as *not important* or *somewhat important*, while 32% were *neutral* about this factor. 59% of respondents noted that it was *not important* to complete placements with friends.

### Strategies to find a CEMS placement

The top three frequently selected strategies used by respondents to find suitable placements were: (i) approaching a practice they knew (93%), (ii) liaising with peers for recommendations (68%), and (iii) searching the Internet (63%). Respondents infrequently used other methods such as: consulting the UCD CEMS past providers list (17%), searching specific veterinary websites (15%), or following up on communications from email or social media posts (11%). Only three respondents referred to UCD notice board posts, the VCI or RCVS register, or the CEMS student handbook. Other strategies identified in an open-ended question (n = 6) included: reviewing student-led message boards mainly based in the United Kingdom, consulting with family friends who were veterinarians, networking with veterinarians at conferences, word of mouth, and finding placements where accommodation could be secured in advance.

### Obstacles to planning CEMS

Participants were asked to select from a list of obstacles they encountered when planning, if any. Table 2 provides the responses from 72 participants. The top two obstacles while planning CEMS were: (i) placements that respondents wanted were already booked (44%), and respondents could not finance placements they wanted (40%). 33% of respondents indicated however, they were able to get all the placements they wanted, which was the third highest selected option from the list. 19% of respondents indicated they took placements in a geographical area they wanted, while 17% selected they took placements they could secure. Seven respondents noted further or reiterated obstacles in the open-ended comment: cost and financing, not receiving email responses from a practice, visa issue, not having a list of potential practices outside of the Republic of Ireland, practice cancelling a placement, finding a practice that would provide opportunities to develop clinical practical skills, requirements for small animal internships post-graduation.

**Table 2:** Obstacles to securing CEMS placements ordered by frequency of selection

Obstacles to CEMS planning	**n**	**% selected**
Placements I wanted were already booked out	32	44%
I could not finance certain placements I wanted	29	40%
Not applicable, I got all the placements I wanted	24	33%
I could not find placements in the geographical area I wanted	14	19%
I took placements that I could secure	12	17%
I did not have enough time to do all the types of placements I wanted	10	14%
I did not get enough placements in specialist/referral practices	9	13%
I was not aware that the timing of placements (i.e. seasonal) would affect my learning experiences	8	11%
I did not know where to find appropriate placements	6	8%
I was not confident enough to contact a veterinary practice	6	8%
I did not get the mix of species I was looking for	4	6%
I did not get enough first opinion only placements	3	4%
I could not find placements on a research project I wanted	3	4%
I did not get enough public health placements	2	3%
I could not find placements in a laboratory I wanted	0	0%

### Financing CEMS placements

Participants were asked to approximate how many placements of their CEMS were financed according to a list of categories. The mean number of placements respondents undertook in locations that were easily accessible and did not involve significant additional costs to attend, for example staying with family or friends, was 6.6 (range 0-12, *n*=72). The mean number of placements that were at a distance where respondents had to find and pay for accommodation was 2.5 (range 0-12, *n*=61). While the mean number of placements that were at a distance and required a loan or money to be borrowed from family or friends to cover costs was 1.5 (range 0-7, *n*=64). Placements that were at a distance and had accommodation costs covered ranged between 0–7, mean was 0.9, *n*=57.

## Learning activities on CEMS

This section of the questionnaire aimed to explore what learning activities students engaged in while on CEMS, results are provided in Table 3. In response to passive learning activities that were primarily associated with observation, for example observing a veterinary practitioner to complete a clinical task, 91% indicated they *almost always* engaged in this type of activity; while 80% indicated they *almost always* observed a team managing a case. The modal response to learning activities associated with assisting practitioners / team to complete a clinical task or manage a clinical case was *sometimes,* however 44% indicated they *almost always* assisted a veterinary practitioner to complete a clinical task, while 41% indicated they *almost always* assisted managed a clinical case. 88% noted they performed clinical skills under direct supervision *sometimes* or *almost always*. The reported frequency of performing clinical skills with supervision at a distance was varied, 40% reported they *rarely* or *every once in a while* engaged in this activity*,* compared to *61%* who indicated they *sometimes or almost always*. Learning activities that are more strongly related to SRL processes varied in frequency of occurrence. As seen in Table 3, when all statements were ranked by weighted mean, respondents' experiences of discussing learning goals with a placement host was the lowest ranked learning activity statement. There was varying frequency of reported engagement with this task, 51% indicated *sometimes* or *every once in a while* and 43% indicated *never* or *rarely*. 43% indicated they *rarely* or *every once in a while* reflected on their previous tasks, while 57% *sometimes* or *almost always* did. There were varying experiences of receiving external feedback from staff members in the clinic and utilising the practices in-house learning resources. 91% of respondents indicated that they *sometimes* or *almost always* asked a member of staff in the veterinary for advice.

**Table 3:** Frequency of learning activities on placements overall. Ranked by weighted mean

Learning Activity	Never (1)	Rarely (2)	Every once in a while (3)	Sometimes (4)	Almost always (5)	Total Responses (n)	Mode	Weighted Mean
Observing a veterinary practitioner completing a clinical task	0%	0%	2%	8%	***91%***	66	5	4.89
Observing a veterinary practitioner / team managing a clinical case	0%	0%	3%	17%	***80%***	66	5	4.77
Assisting a veterinary practitioner to complete a clinical task	0%	0%	8%	***49%***	44%	66	4	4.36
Assisting a veterinary practitioner / team to manage a clinical case	0%	0%	12%	***47%***	41%	66	4	4.29
Asking a member of staff in the veterinary practice for advice	0%	5%	5%	***48%***	43%	65	4	4.29
Discussing a clinical case with the veterinary team	0%	2%	14%	***42%***	***42%***	66	4^a^	4.26
Performing clinical skills under direct supervision	0%	2%	11%	***62%***	26%	66	4	4.12
Receiving feedback on my clinical tasks from a member of staff in the veterinary practice	5%	8%	32%	***38%***	18%	66	4	3.58
Reflecting on my previous tasks	0%	15%	28%	***40%***	17%	65	4	3.58
Performing clinical skills with supervision at a distance	0%	20%	20%	***55%***	6%	66	4	3.47
Acquiring new information from the practice learning resources (e.g. literature, books etc.)	5%	11%	30%	***44%***	11%	66	3	3.45
Using the practice’s training materials or any documentation relating to protocols or standard operating procedures	14%	11%	26%	***36%***	14%	66	3	3.26
Discussing my learning goals with my supervisor	11%	32%	24%	***27%***	6%	66	2	2.86

Mode highlighted in bold and italics. ^a^indicates there was more than one mode

## Assessment of learning experiences on CEMS

Participants were asked to self-assess their learning experiences on CEMS and to indicate how it supported the development of their professional attributes, clinical skills and understanding of veterinary professional working life, results are shown in Table 4, which are ranked by weighted mean. The majority of respondents *agreed* or *strongly agreed* that CEMS supported the development of all competencies except for the preparation of accurate medicals where the modal response was *strongly disagree*. Both statements in the questionnaire that related to CEMS supporting an appreciation of veterinary professional daily life and experience of general practice / primary care settings were rated very highly, the majority of respondents either *agreed* or *strongly agreed* with these statements. 65% of respondents *agreed* that CEMS helped them to recognise their limitations and to identify where to seek support. 73% *agreed* that CEMS supported them to understand situations that required referral. There was variability between specific learning experiences in terms of learning how veterinary practices are run as a business and history taking skills.

**Table 4:** Self-assessment of learning experiences that supported the development of competencies. Ranked by weighted mean

Learning Experiences	Strongly disagree (1)	Disagree (2)	Neither agree or disagree (3)	Agree (4)	Strongly agree (5)	Total Responses (n)	Mode	Weighted Mean
CEMS showed me how different veterinary practices work on a day-to-day basis	0%	0%	0%	46%	***54%***	69	5	4.54
CEMS gave me opportunities to apply my veterinary knowledge to a real-life context	0%	0%	1%	***58%***	41%	69	4	4.39
CEMS gave me experience of working within real-life constraints that I had not experienced at UCD (e.g., 10-min consults, limited treatment options, limited availability of equipment)	0%	1%	9%	42%	***48%***	69	5	4.36
CEMS helped me develop my animal handling and restraining skills	0%	3%	3%	***58%***	36%	69	4	4.28
CEMS gave me opportunities to develop my communication skills with veterinarians, nurses and practice owners	0%	1%	7%	***55%***	36%	69	4	4.26
CEMS gave me opportunities to work as part of a veterinary team	0%	6%	7%	***54%***	33%	69	4	4.14
CEMS gave me a greater insight into the differences and requirements between 1st opinion and referral work	0%	3%	12%	***61%***	25%	69	4	4.07
CEMS helped me develop my clinical examination skills	1%	6%	9%	***55%***	29%	69	4	4.04
CEMS gave me experiences to develop my clinical problem-solving skills	0%	3%	16%	***59%***	22%	69	4	4.00
CEMS gave me opportunities to communicate with clients	1%	12%	7%	***48%***	32%	69	4	3.97
CEMS helped me recognise my limitations and to know where to seek advice and assistance	0%	3%	16%	***65%***	16%	69	4	3.94
CEMS helped me to identify situations that should be referred	0%	6%	12%	***73%***	10%	69	4	3.87
CEMS enabled me to see how ethical and legal responsibilities apply in real-life situations	0%	6%	17%	***61%***	16%	69	4	3.87
CEMS gave me a good understanding of how veterinary practices are run as a business	0%	17%	13%	***49%***	20%	69	4	3.72
CEMS brought me into contact with species or areas of veterinary work that I had not dealt with at UCD	4%	22%	12%	***39%***	23%	69	4	3.55
CEMS helped me develop my history taking skills	7%	12%	25%	***42%***	15%	69	4	3.45
CEMS helped me to prepare accurate medical records	4%	***39%***	28%	25%	4%	69	2	2.86

Mode highlighted in bold and italics

## Reflections of CEMS

Participants were asked to rate six statements that considered their overall reflections of CEMS and if alternative educational experiences should be offered. Three statements were rated highly by respondents who *agreed* or *strongly agreed* that CEMS is an essential part of the undergraduate veterinary degree program (92%, n = 65); CEMS supported their decisions regarding the type of work they wished to undertake after graduation (88%, n = 65); and CEMS made them more employable and prepared for work (87%, n = 65). The majority of respondents either *disagreed* or *strongly disagreed* with two further statements suggesting possible alternatives for CEMS weeks: replacing all CEMS weeks by increasing intramural rotations in UCD (85%, n = 65), or replacing some CEMS week by increasing time spent in the UCD clinical skills laboratory (68%, n = 64). Responses varied regarding the suggestion of replacing some CEMS weeks by increasing the number of intramural rotations, 57% (n = 64) *strongly disagreeing* or *disagreeing* with this statement while 34% (n = 64) *agreed* or *strongly agreed*.

### Overall planning experience

Respondents were asked to reflect on their overall planning experience and to select the statement that best describes their experience, there were 65 responses to this question. 65% of respondents noted they completed their CEMS with minor problems that were easily resolved. 42% of respondents noted they were able to find and complete the placements they needed. 2% indicated they completed their CEMS but it required a significant amount of research and effort. One respondent noted it was a real struggle to find and book placements.

### Recommendations to improve CEMS

42 respondents provided comments highlighting their recommendations to improve CEMS for both students and placement hosts. Two themes from the thematic analysis were developed: planning supports, expectations and formalising learning on CEMS. Quotes have been included to illustrate the key ideas of the themes or nuances.

### Planning supports

Planning of CEMS was viewed by respondents to be financially challenging given that it has to be completed during non-academic trimester weeks. For some this impacted their ability to work in order to support their studies. One respondent highlighted this caused them some stress. A funding scheme was suggested.

*“Provide financial support for students in order to undertake CEMS, even if that is in the form of accommodation, as in human medical schools. Due to CEMS, we are generally unable to work a paying job, which further compounds the high cost of veterinary school.*” (2017, GE)

One respondent commented that giving extra time for CEMS during the academic trimester, in particular during the lambing season would be helpful.

*“I know its difficult to arrange with UCD but maybe give vet students time off at spring for calving season.”* (2017, MVB)

Respondents suggested the development of a “*Student to student practice and experience feedback scheme*” (2017 MVB) for CEMS to support other students to source suitable CEMS placements. This information would include the type of practice, learning opportunities available and key contact. It was suggested this may involve veterinary practices nominating themselves and providing information for the scheme.

### Expectations and formalising learning on CEMS

Respondents highlighted that CEMS experiences can be variable in terms of practical clinical skills development and level of interaction with the veterinary team. Suggestions were made to formalise learning by including a specific list of skills to undertake, procedures to be completed and a possible move to a distributed model of work placements similar to that implemented in the UCD Veterinary Nursing programme. Respondents viewed greater communication between placement hosts and UCD would assist student learning on CEMS. One respondent highlighted that additional clinical skills practicals conducted in UCD in advance of CEMS would be supportive.

*“Practices vary a lot in the way they teach students and how much they let them do, and as a result, I think my overall experience with CEMS was a bit of a mixed bag. Some placements were excellent in regards to hand-on work and interaction/discussion with the vets, and at others I was no more than an observer and not even allowed to help restrain an animal. Unless you got recommendation from another student who had done CEMS at the practice, you had very little idea of what to expect at each placement.” (2018, GE).*

One respondent suggested that students should find a suitable placement host where they would do the majority of their CEMS weeks. This would allow them time to build trust with the practice team.

*“Ensure that students have a good base practice that will support them allow them to learn. Encourage students to spend time in what they think will be their base practice for CEMS prior to starting their actual CEMS so that they get to know the practice and that the vets get to know the students and to trust them.” (2018, MVB)*

## Discussion

The aim of this study was to explore how final year students’ in UCD planned, learned and reflected on their CEMS experiences. The findings of this study suggest that students highly value CEMS as a co-curricular learning experience to their undergraduate veterinary medicine programme, and that CEMS experiences aided their future career choices and their preparation for the workplace. Students were motivated to plan CEMS placements that would support or guide future career goals, however, the cost of financing CEMS placements played a key role in planning decisions. The data presented indicate that students did not frequently engage in learning activities such as discussing their learning goals with their placement supervisor, receiving external feedback or reflecting on their learning, which are activities more strongly related with SRL processes. Students self-assessed that the majority of day-one competencies were developed through their CEMS experiences and overall any problems planning their CEMS were easily resolved.

Effective self-regulated learners are able to set personal learning goals that will guide their decision making and subsequently their learning, this process is influenced by the individual's motivation and self-efficacy [34]. The survey highlighted that students are motivated by placements that would be beneficial to a future career goal or possible future employment. The student activity records for the GE programme indicate they completed the majority of their placements in small animal practices. As many on this programme are international students [35] and have worked as veterinary technicians in clinical practice, these prior experiences may have already focused their career aspirations and subsequent CEMS placement plans and goals. Career motivations are important in light of the results of a previous study conducted in the UK in 2018 of veterinary students and graduates by the *Association of Veterinary Students*. They noted that participants agreed that CEMS assisted their career preparation and 58% of graduates indicated they received an employment offer from one of their EMS placement hosts [36].

Affordability of a CEMS placement was regarded by 83% of the survey respondents to be a key motivational driver for CEMS choices, and financial barriers were cited as the second highest obstacle to planning. The majority of students reported in the survey that they choose to complete their placements in a location that did not incur significant costs (accommodation, transportation). 93% of students indicated they approached a placement they knew; further research would be required to ascertain why, but it may be that the practice was local to them and therefore costs to attend could be reduced. Financial concerns surrounding CEMS have been further reported by the RCVS in their consultation with stakeholders [37] and in their 2014 student survey from which our questionnaire was adapted [33]. Our survey findings indicated that students would be supportive of a funding scheme to support CEMS. While some sources currently exist in the UK [38, 39], accessing funding may be a challenge for students. Following a recent stakeholder consultation by the RCVS a new EMS policy was published in 2022 that aims to improve how students can plan their CEMS and provides greater flexibility for students to set individualised learning goals [40]. The number of CEMS weeks will be reduced to help offset the financial burden of EMS as the demand for CEMS placements continues to grow in parallel to the increasing number of veterinary students as new veterinary schools open. At the time of writing there is a shortage of accommodation in the rental market and a cost of living crisis in Ireland that has been widely reported in the national media [41]. Furthermore, there are ongoing concerns regarding the cost of Higher Education in Ireland that the Irish government is seeking to address through a ‘*Funding the Future*’ policy that aims to provide a model of Higher Education that is accessible to all [42]. Students are facing significant challenges to finance their studies, while not explored in this survey, these socio-economic factors may play an increasing role affecting student goals and motivations in planning their CEMS.

The survey highlighted that students did not consistently discuss their learning goals with their CEMS placement hosts, an activity that would support SRL processes. Sitzman and Ely [13] highlight in their systematic review of SRL in a work-related training that goal level, persistence, effort and self-efficacy were constructs that had the strongest influence on learning outcomes. The guidelines to support student learning on CEMS by the RCVS encourage students to develop learning objectives and discuss these with their placement hosts [43], an approach encouraged by UCD and by the *Association of Veterinary Students* [44]. If these discussions were to occur more frequently it may help to manage learning expectations and facilitate learning opportunities that are realistic for the student and the activities of the practice. Our study did not explore why students did not discuss their goals with their placement hosts more frequently and what challenges they may have faced in their efforts. Further research is recommended to explore how and why students choose their learning goals for CEMS, what difficulties they may have experienced discussing these with their placement host, and how learning goals were utilised to regulate their learning in the clinical environment.

Developing effective learning strategies in the context of the clinical environment is important for students where efficient patient care is the priority. Strategies for learning will differ from those implemented in formalised academic contexts. Instead students must be able to recognise potential learning opportunities and be able to act [10]. Berkhout [45] identified factors that influence students’ self-regulation in the clinical context: (i) the opportunities received or created, which are affected by multiple attributes including facilities available, interaction with clients, cases available, engagement with staff, workplace dynamics; (ii) level of autonomy experienced, and (iii) anticipated outcomes. Students can also struggle with confidence, understanding their role and what was expected of them by clinicians when they transitioned to learning in the clinical workplace [11]. The survey results highlight that students ‘*almost always*’ engaged in learning activities that were passive and observational. While our study did not explore the factors that inhibited greater active participation with the clinical team on CEMS, it may be that contextual factors identified in previous research on SRL in the clinical workplace and role expectations impacted students’ ability to engage more actively. Survey data indicated that students did not frequently reflect on their learning activities on CEMS and qualitative data from the survey suggest that students sought a formalised approach to their learning on CEMS, through the introduction of a specific list of tasks for practices to teach. It may be that students need guidance to develop their reflective learning skills [46] to maximise their learning from observation in the clinical environment, and to develop skills to identify alternative informal learning opportunities available. Educational theories of experiential learning [47] and zone of proximal development [48] can guide educators to develop and scaffold educational interventions to support students to actively analyse observational clinical experiences encountered with veterinary professionals on CEMS and to extract meaning to further their knowledge [49-52]. Developing these reflective learning skills will be important for students on CEMS, as observation is likely to be a core activity when students commence their placements.

## Future work and study limitations

Students overall acknowledged that CEMS contributed to the development of professional skills, clinical skills and veterinary knowledge, as well as providing experiences of day-to-day veterinary life. The student learning experience on CEMS however can be improved. We recommend that there is a need to design and develop educational interventions that support students to self-regulate their learning in the clinical context and develop learning strategies to avail of informal learning opportunities and to reflect on learning from CEMS. Academic advising and mentoring programmes [53] have shown positive outcomes in supporting students' career goals, motivation, lifelong learning skills and reflective learning. Further enhancements to the design of the veterinary medicine curriculum could aid students' preparation and transition to WPL, for example increased clinical skills development prior to taking CEMS, and professional development activities to guide students in navigating the complexities of learning in the workplace and their interaction with daily practice. Further collaboration with CEMS placement hosts may also prove beneficial by providing training and resources to support their role with students. This is a complex issue that will require input and collaboration from all stakeholders. Future research should explore the effectiveness of such interventions to improve student’s SRL practices in the clinical workplace and to investigate SRL sub-processes for example, setting appropriate learning goals, choosing and implementing effective learning strategies, and reflective learning in the context of informal learning on CEMS.

The single-institution design of this study is a limitation as findings cannot be generalised to other workplace settings or other educational health professional contexts, however small-scale social research projects using an exploratory sample using non-probability methods can provide insights [54]. The response rate to the questionnaire was low (23% in 2017 and 42% in 2018), which is below the 50% target for each sample the study had aimed for. This may be attributed to the timing of the questionnaire invitation and its voluntary nature. Communication was sent to students in March of their final-year, where students are completing intramural placements and will have been under time pressure for upcoming final examinations. It is therefore possible that those who responded to the questionnaire may have been particularly motivated to share positive or negative feedback about CEMS which may have resulted in the data being skewed by the respondent population. A further limitation of the study is the use of self-reporting questionnaires for investigating SRL experiences. Rowers et al. [55] highlight that this approach may provide insights globally into students' own level SRL versus more specific SRL processes where behaviour measures may be more accurate. Our survey sought to provide an overarching view of the planning, learning and reflective activities for CEMS by students in an Irish higher education institution. These activities were analysed through the theoretical lens of SRL, a previously unexplored area.

## Conclusions

This study contributes to the veterinary educational literature by providing evidence of student engagement with SRL activities in an Irish veterinary medicine programme. The study found that students focused their CEMS placements in small animal, production animal or mixed practices. Students highly valued CEMS as part of their veterinary education and engaged in a range of activities to regulate their learning and attain their goals. Data provided insights into the planning and learning challenges students faced which agrees with other reported studies in the medical educational literature. This data can support a greater understanding of the veterinary student WPL experience in the context of CEMS and highlights an opportunity to develop educational supports to enhance their learning.

## Data Availability

The datasets generated and analysed during the current study are not publicly available as the participants of this study did not give written consent for their data to be shared publicly, but are available from the corresponding author on reasonable request.

## Abbreviations

UCD: University College Dublin
CEMS: Clinical extramural studies
MVB: Five-year undergraduate veterinary medicine programme
GE: Four-year graduate entry veterinary medicine programme
SRL: Self-regulated learning
WPL: Workplace learning
RCVS: The Royal College of Veterinary Surgeons
